# The Evaluation of Physio-Mechanical and Tribological Characterization of Friction Composites Reinforced by Waste Corn Stalk

**DOI:** 10.3390/ma11060901

**Published:** 2018-05-27

**Authors:** Yunhai Ma, Siyang Wu, Jian Zhuang, Jin Tong, Yang Xiao, Hongyan Qi

**Affiliations:** 1 State key laboratory of automotive simulation and control, Jilin University, Changchun 130022, China; myh@jlu.edu.cn (Y.M.); siyangwu@outlook.com (S.W.); jtong@jlu.edu.cn (J.T.); xiaoyang_jlu@163.com (Y.X.); m15543610031@163.com (H.Q.); 2 Key Laboratory of Bionic Engineering, Ministry of Education, Jilin University, Changchun 130022, China; 3 College of Biological and Agricultural Engineering, Jilin University, Changchun 130022, China; 4 State Key Laboratory of Automotive Safety and Energy, Tsinghua University, Beijing 100084, China

**Keywords:** corn stalk fiber, friction composite, friction and wear, worn surface morphology

## Abstract

This paper addressed the potential use of fibers from waste corn stalk as reinforcing materials in friction composites. The friction composites with different contents of corn stalk fibers were prepared, and their tribological and physio-mechanical behaviors were characterized. It was found that the incorporation of corn stalk fibers had a positive effect on the friction coefficients and wear rates of friction composites. Based on comparisons of the overall performance, FC-6 (containing 6 wt % corn stalk fibers) was selected as the best performing specimen. The fade ratio of specimen FC-6 was 7.8% and its recovery ratio was 106.5%, indicating excellent fade resistance and recovery behaviors. The wear rate of specimen FC-6 was the lowest (0.427 × 10^−7^ mm^3^ (N·mm)^−1^ at 350 °C) among all tested composites. Furthermore, worn surface morphology was characterized by scanning electron microscopy and confocal laser scanning microscopy. The results revealed that the satisfactory wear resistance performances were associated with the secondary plateaus formed on the worn surfaces. This research was contributive to the environmentally-friendly application of waste corn stalk.

## 1. Introduction

Friction composites are commonly used in transmission and brake systems for safe rapid deceleration and immobilization of various vehicles and instruments [1,2,3]. Friction composites should possess a certain set of outstanding properties, including a moderate friction coefficient, high heat fading resistance and recovery, no or less noise and vibration, and low wear rate under different operating environments [4,5,6,7]. For this reason, friction composites normally contain more than ten ingredients, which are separated into four prime classes of reinforcing fibers, friction modifiers, binder resins, and space fillers [8,9,10]. Among them, reinforcing fibers have a pivotal role to play in deciding the tribological and mechanical properties of friction materials. Ceramic, organic, and metallic fibers are mainly used as substitutes for traditional asbestos fibers in friction composites [11,12]. 

Among the diverse fibers available for friction composites, natural fibers have drawn much attention as reinforcing materials because of their environmental friendliness, renewability, low density, low costs, excellent acoustic insulating properties as well as their satisfactory mechanical performances [13,14]. In recent years, many studies investigated the influences of natural fibers on the tribological characteristics of friction composites [15]. Chand et al. [16] developed polyester composites reinforced by jute fibers and evaluated the effects of applied load and fiber orientation on friction and sliding wear properties. The results showed that the friction coefficients declined with the rise of applied load and the wear resistance maximized under normal orientation, indicating these reinforced polyester composites may have potential application as friction materials in environmentally-friendly brake pads. Bajpai et al. [17] reported the influence of nettle, grewia optiva and sisal fibers on the wear and frictional behaviors of poly lactic acid (PLA) composites. This study revealed that the addition of these natural fibers remarkably enhanced the wear performance of PLA composites, as the specific wear rate and friction coefficients of the composites were reduced by 10–44% and more than 70%, respectively, in comparison with neat PLA. Nirmal et al. [18] prepared polyester composites reinforced by treated betelnut fibers and studied their mechanical and tribological behaviors under dry/wet sliding conditions. The study suggested that the friction coefficients and average wear rates under the wet sliding condition dropped significantly by about 95% and 54% respectively compared with under dry conditions, and the wear resistance was improved under an anti-parallel orientation to the sliding surface. Fu et al. [19] evaluated the tribological characteristics of phenolic resin-based friction composites containing treated flax fibers under dry contact conditions. The study found that the introduction of flax fibers into resin substrate stabilized the friction coefficients and improved wear resistance, indicating that flax fibers were an ideal substitute for asbestos in brake pads. Given the above advantages and chances of natural fibers, further research is needed to explore and evaluate the tribological behaviors of other types of natural fibers.

Corn is one of the most productive cereals in China, especially in the northeast area. It is estimated that approximately 0.23 billion tons of corn stalks are generated annually as agricultural by-products [20,21,22]. However, after the harvest, most corn stalks are left on the field or burned, which leads to a waste of resources and environmental degradation [23,24]. Hence, it is essential to seek an effective and environmentally friendly way to improve the utilization value of corn stalks.

This work was aimed to study the feasibility of applying fibers obtained from corn stalks as reinforcement fibers to the manufacture of friction composites. For this purpose, five types of friction composites were fabricated with corn stalk fiber content of 0 wt %, 2 wt %, 4 wt %, 6 wt %, 8 wt % respectively. Then their physio-mechanical and tribological behaviors were characterized and evaluated systematically. Furthermore, the wear mechanisms of the corn stalk fiber-reinforced friction composites were explored and analyzed based on worn surface morphologies.

## 2. Materials and Methods 

### 2.1. Preparation of Corn Stalk Fibers 

The degree of interface adhesion between fibers and matrix affects both the physio-mechanical behaviors of natural fibers-reinforced friction composites and the reinforcing efficiency of the fibers [25,26]. Thus, in the preparation of friction composites reinforced by natural fibers, the indispensable step to improve the fiber-matrix interface bonding is surface modification (e.g., alkali treatment, benzoylation treatment, acetylation treatment, silane treatment and electric discharge treatment) [27,28,29]. In this study, alkali treatment was used as fiber modification to increase the compatibility with the matrix. 

Corn stalks obtained from a local farm in Changchun, China, were naturally air-dried for a few days and then separated into rinds and piths. The rinds were ground into 3–4 mm long fibers, which were surface-treated as described below. In the alkalization, the fibers were immersed in 1% aqueous NaOH solution at 30 °C for about 20 min and rinsed with distilled water until turning pH 7. Finally, excessive solvent and moisture were removed from the corn stalk fibers after treatment in a ZK350S vacuum drying oven (Sanshui, Tianjin, China) at 90 °C for 4 h [30]. 

### 2.2. Fabrication of Friction Composites

**Figure 1:**
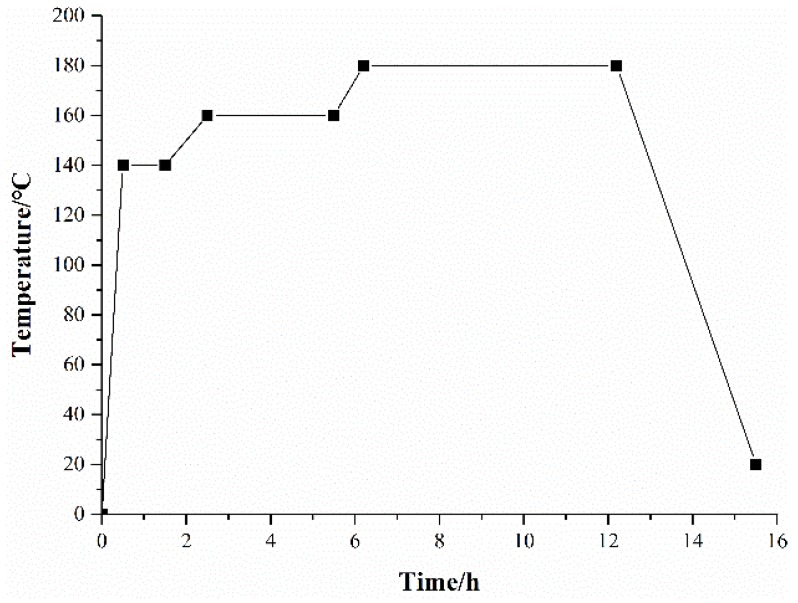
Heat-treatment process of the composites.

The detailed compositions of the friction materials (in wt %) are presented in Table 1. The five types of friction composites were numbered as FC-0, FC-2, FC-4, FC-6, FC-8, respectively, according to the content of corn stalk fibers. The friction composites were prepared via compression molding. Firstly, the raw materials were mixed thoroughly in a JF805R electrical blender (Wangda, Changchun, China) for 8 min. The uniform mixture was then molded for 30 min at 160 °C under 45 MPa by using a JFY50 hot compression machine (Wangda, Changchun, China). Three intermittent ‘breathings’ were required in the process of hot pressing to release volatiles. The prepared friction composites were subsequently heat-treated in an oven to remove the remaining stress, which involved three phases (Figure 1): 140 °C × 1 h, 160 °C × 3 h, and 180 °C × 6 h. Finally, the friction composites were air-cooled to room temperature and machined into specimens of 25 × 25 × 6 mm^3^.

### 2.3. Testing Methods and Equipment

The density of each friction composite was measured on an MP-5002 electronic balance (Junda, Shenzhen, China) following the Archimedes drainage approach. The hardness was tested using an HRSS-150 Rockwell hardness tester (Sannuo, Shenzhen, China) as per the *Test method of Rockwell hardness for friction materials* (GB/T 5766-2007). The impact strength was detected on an XJ-40A impact testing machine (Jianyi, Shanghai, China) based on the *Test Method for Tensile-Impact of Plastics* (GB/T 13525-92). Each specimen was tested in quintuplicate to minimize the error.

**Figure 2:**
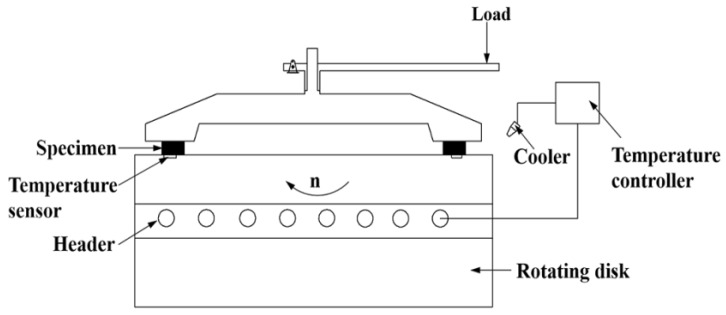
Schematic diagram of friction testing machine.

Tribological performances of the friction composites were evaluated on a JF150D-II constant-speed friction instrument (Wangda, Changchun, China) as per *Brake Linings for Automobiles* (GB/T 5763-2008), with a schematic diagram showed in Figure 2. The specimens were pressed against the surface of the rotating disk by pressurizing device under a certain load condition. The frictional force and temperature during the test were detected by the tension-compression sensor and temperature sensor respectively. The temperature was controlled and stabilized at the set value through the heating system and cooling system. An HT250 cast iron disk with hardness of 180–220 HB was used as the mating plate. The friction and wear tests consisted of two parts of fade tests and recovery tests. Five parallel tests were carried out to reduce the data scattering.

In the fade tests and recovery tests, the temperature was changed from 100 °C to 350 °C and from 300 °C to 100 °C, respectively, and the disk was rotated 5000 and 7500 revolutions, respectively, before measurement of thickness change and weight loss. Moreover, the rotating speed and contact pressure were set at 480 rpm and 0.98 MPa, respectively in all tests. 

The friction coefficient was automatically recorded through the computer attached to the friction tester. The wear rate ΔW was determined by the following equation [31,32]:

(1) $$\Delta W=\frac{1}{2\pi R}\times \frac{A}{N}\times \frac{h_{1}-h_{2}}{f}$$

where *R* (=150 mm) is the horizontal distance from a friction specimen to the counterpart disk center; *A* (=625 mm^2^) is the area of the friction specimen; *N* (=5000) is the revolutions of the disk; *h*_1_ and *h*_2_ are the average thicknesses of the friction specimen before and after tests, respectively (mm); *f* is the mean friction force during tests (N).

The fade ratio *F* and recovery ratio *R* were defined by the following equations [33,34]:

(2) $$F=\frac{\mu_{F100^{{}^{\circ}}C}-\mu_{F350^{{}^{\circ}}C}}{\mu_{F100^{{}^{\circ}}C}}\times 100\%$$

(3) $$R=\frac{\mu_{R100^{{}^{\circ}}C}}{\mu_{F100^{{}^{\circ}}C}}\times 100\%$$

where *μ*_F100°C_ and *μ*_F350°C_ are the friction coefficients with the temperature rise to 100 °C and 350 °C during fade tests, respectively; *μ*_R100°C_ is the friction coefficient with the temperature declined to 100 °C during recovery tests. 

After the friction and wear tests, the worn surface morphology of each specimen was observed by an EVO-18 scanning electron microscope (SEM, ZEISS, Jena, Germany) at 20 kV, and 3D profiles and surface roughness were detected on a LEXT OLS3000 confocal laser scanning microscope (CLSM, OLYMPUS, Beijing, China) as per *Geometrical Product Specifications(GPS)—Surface texture—Profile method—Surface roughness—Terminology—Measurement of surface roughness parameters* (GB/T 7220-2004).

## 3. Results and Discussion

### 3.1. Surface Morphology of Corn Stalk Fibers

**Figure 3:**
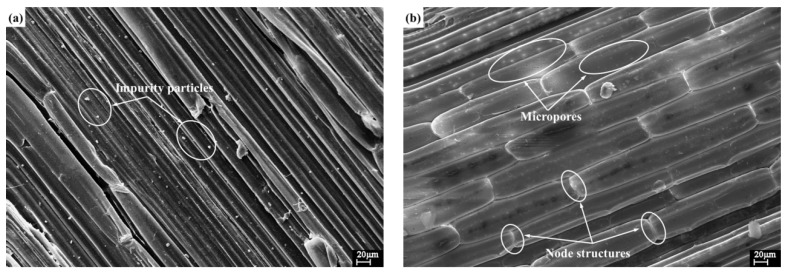
Micrographs of (**a**) raw and (**b**) treated corn stalk fibers.

Surface morphology of the raw and treated corn stalk fibers is shown in Figure 3. Clearly, the raw fibers presented smooth outer surfaces with some impurity particles (Figure 3a). After alkali treatment, the surfaces became cleaner and contained a large number of node structures and micropores (Figure 3b), indicating the corn stalk fibers were significantly modified. These changes may be ascribed to the removal of natural and artificial impurities (e.g., lignin, wax, pectin and oils) and the increased amount of exposed cellulose on the fiber surfaces, which could improve the fiber-matrix interfacial adhesion [25,35].

### 3.2. Physio-Mechanical Performances

The physio-mechanical performances of friction composites are modestly associated with the reliability and security of vehicle operation. The density, hardness, and impact strength of the composites are summarized in Table 2. The densities of these composites decreased with the increasing content of corn stalk fibers. The density of specimen FC-0 was the largest and that of specimen FC-8 was the smallest among all composites. The hardness of the composites showed a similar tendency with increase in content of corn stalk fibers, as it was maximized in specimen F-0 and minimized in specimen FC-8. However, no obvious variation trend was observed in impact strength. The impact strength of specimen FC-4 was the highest, while specimens FC-2 and FC-8 had the lowest impact strength in all tested composites. This may be because excellent interface adhesion between the reinforcing fibers and the matrix could enhance the impact resistance of friction composites to some degree. It was indicated that the addition of corn stalk fibers can enhance the physio-mechanical properties of the friction composites. This is in agreement with the results of the previous report [36].

### 3.3. Friction and Wear Behaviors

**Figure 4:**
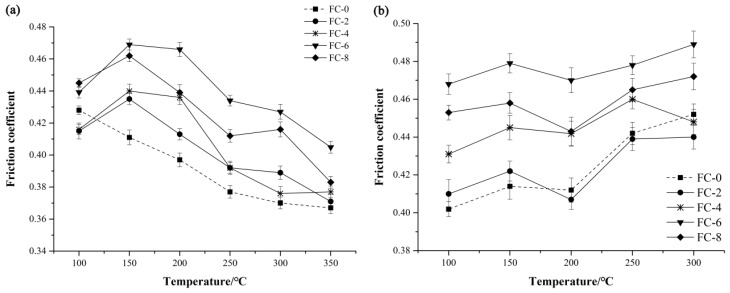
Variation in friction coefficient of the friction specimens: (**a**) fade test and (**b**) recovery test.

Variations in the friction coefficients of the five composites during the fade and recovery tests are presented in Figure 4. The friction coefficient of specimen FC-0 decreased with the temperature rise, while the variant trends of the other four composites were slightly different (Figure 4a). The friction coefficients of the composites incorporated with corn stalk fibers initially increased with the temperature rise from 100 °C to 150 °C and then decreased from 150 °C to 350 °C These changes can be explained by the fact that during the initial phase of the test (100–150 °C), the fibers and some hard particles were exposed to the worn surface due to the removal of the matrix and soft materials, then the wear debris gathered around the nucleation sites formed from protruding fibers, followed by the generation of the third body wear under the action of frictional force and normal pressure, which resulted in the increase of the friction coefficients [2]. When the temperature was higher than 160 °C, the lignin in the fibers began to decompose and the fibers gradually carbonized [37], then some carbon powder appeared on the friction surface, which had a certain lubrication effect, thus leading to the decline of the friction coefficients [36]. Moreover, the sheer strength of friction composites declined with the rise of surface temperature, which also resulted in the decrease of the friction coefficient. Anyway, the friction coefficients at each test temperature were in conformity with Chinese national standards. The friction coefficients of the five friction composites decreased slowly at 250 °C or above, which can be ascribed to the thermal relaxing and degradation of phenolic resins at elevated temperatures during the tests [32]. 

In general, the addition of corn stalk fibers improved the friction behaviors at tested conditions barring the 100 °C case. The reason for this phenomenon could be that when the resin matrix was worn off, the reinforcing fibers were exposed to the friction surfaces of the composites and scraped the mating plate, which was transformed to frictional output. This is consistent with the previous report [38]. Among these tested specimens, the specimen FC-6 showed the highest friction coefficient except for the case at 100 °C where the specimen FC-8 showed a little higher friction coefficient. 

During the recovery tests (Figure 4b), the friction coefficients of the composites decreased first with the temperature ranging from 300 °C to 200 °C, and then increased from 200 °C to 150 °C, and finally declined with the temperature varying from 150 °C to 100 °C. On the whole, the variation of friction coefficients was relatively stable, and it fluctuated between 0.402 and 0.489. The recovery fluctuation was one of the major affection factors for the performance of automotive braking.

**Figure 5:**
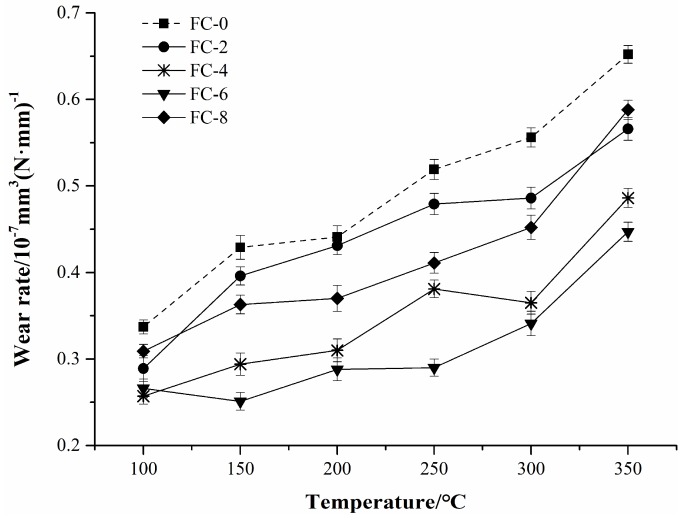
Wear rates of the friction specimens.

The variations in wear rates of the friction composites with test temperatures are illustrated in Figure 5. It can be seen clearly that the wear rates were dramatically affected by temperature and increased with the temperature rise for all composites. The reason for this behavior may be that the phenolic resin gradually began to soften and decompose as test temperatures rise, causing a decrease in interface binding force between the composite matrix and fillers. As a result, the fillers were loosened and debonded from the matrix, which increased the wear rates of the composites. This is in accordance with the previous study results [39,40]. 

Generally, the incorporation of corn stalk fibers enhanced the wear behaviors of the composites, as the wear rates decreased first and then increased with increasing fiber content. It was indicated that there was an optimum fiber content in the formula of friction composites [6]. Among all tested specimens, the wear rate maximized to 0.632 × 10^−7^ mm^3^ (N·mm)^−1^ at 350 °C in specimen FC-0, whereas it minimized to 0.427 × 10^−7^ mm^3^ (N·mm)^−1^ at 350 °C in specimen FC-6 except for the case between 100 °C and 150 °C where the wear rate of specimen FC-4 was a bit lower. This observation suggested that 6 wt % of corn stalk fibers was the optimum dosage for the wear performance of friction composites. A dosage beyond 6 wt % might induce fiber accumulation and uneven distribution in the composite matrix, which would lead to a decline in wear resistance.

### 3.4. Fade Resistance and Recovery Properties

**Figure 6:**
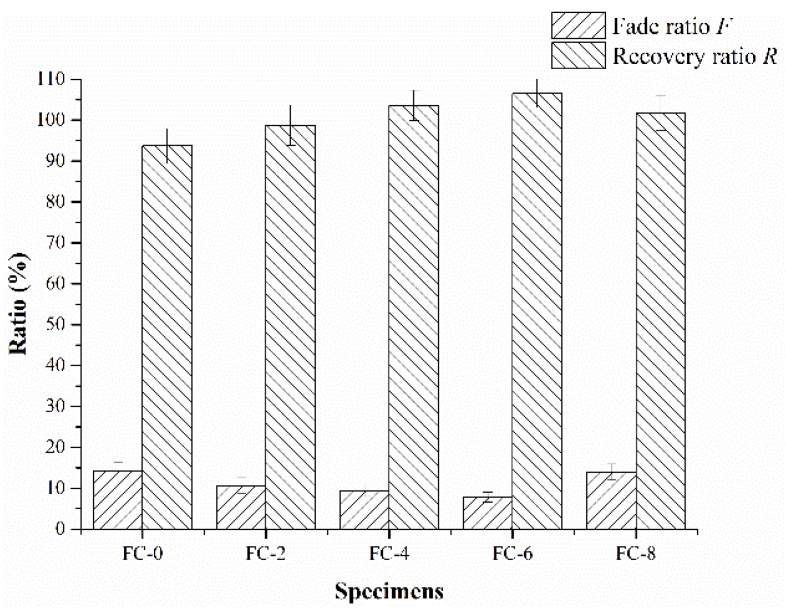
Fade ratios and recovery ratios of the friction composites.

The fade resistance and recovery behaviors are of critical importance in the performance assessment of friction composites, and they can influence braking reliability and effectiveness during the braking process [33]. The friction coefficients decreased gradually with a temperature rise and recovered after a temperature reduction, which were referred to as fade and recovery phenomena, respectively [34]. Fade ratios and recovery ratios were the main parameters for evaluating the friction stability of friction composites and quantitatively characterizing the fluctuations of the friction coefficients. The fade and recovery behaviors of the five friction composites are illustrated in Figure 6. It can be seen clearly that the composites added with corn stalk fibers showed improved fade and recovery behaviors during the tests. The fade ratios of the friction specimens ranked in the order of FC-0 > FC-8 > FC-2 > FC-4 > FC-6, while the order of recovery ratios was FC-6 > FC-4 > FC-8 > FC-2 > FC-0. In particular, the specimen FC-6 had the fade ratio of 7.8% and recovery ratio of 106.5%, indicating it behaved well in fade resistance and recovery. However, the fade ratio of specimen FC-0 was 14.3% and the recovery ratio was 93.6%, indicating its fade and recovery properties were the worst of the five composites.

### 3.5. Analysis of Worn Surface

The tribological performances of friction materials are closely associated with their worn surface morphological properties (e.g., wear debris, primary and secondary plateaus, microcracks and cavities) [31,41,42]. Surface morphology research of friction composites have been reported as an effective tool to interpret the results of tribological behavior analysis and explain the wear mechanisms [43].

**Figure 7:**
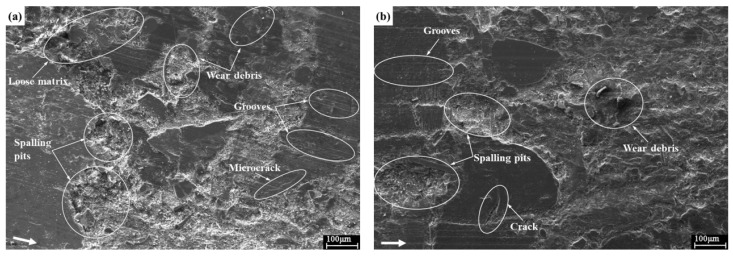

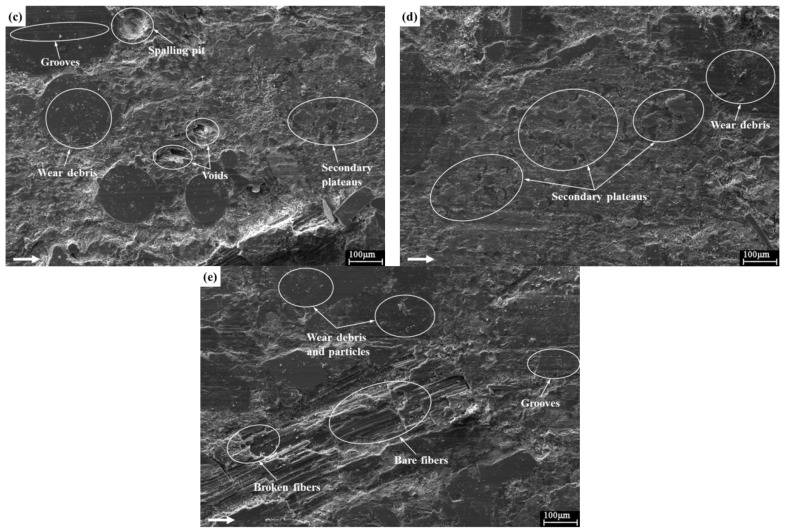
Worn surface morphology of (**a**) FC-0; (**b**) FC-2; (**c**) FC-4; (**d**) FC-6; (**e**) FC-8.

In the present study, the worn surface morphology analysis of friction composites was performed by SEM. The typical worn surfaces of the five friction composites at 350 °C are presented in Figure 7a–e. Specifically, the worn surface of the specimen FC-0 was very rough with severe damage and massive destruction (Figure 7a). A number of fine wear debris and hard particles along with large spalling pits presented on the surface, and loose matrix, microcracks, and a lot of grooves were also evident, which corresponded to high wear rate of specimen FC-0. In general, the thermal relaxation and degradation of resin caused matrix loosening and then generated a lot of wear debris and abrasive particles under the action of friction force. Some of these debris and particles were embedded and removed on the worn surface, and then shallow grooves parallel to the sliding direction appeared on the surface of the specimen, which were all typical of abrasive wear. Meanwhile, large flake debris detached from the surface under the sheer force, and spalling pits presented on the matrix surface, which might be the main cause of adhesive wear. Moreover, owing to varying thermal expansion rates in different regions on the friction surface layer, microcracks appeared on the worn surface of the composite under unstable pressure and temperature field, which could be responsible for the fatigue wear [44]. Hence, the main wear mechanisms of the specimen FC-0 were abrasive wear, adhesive wear and fatigue wear.

As illustrated in Figure 7b–e, the worn surfaces of specimens FC-2, FC-4, FC-6 and FC-8 were relatively smooth in comparison with specimen FC-0, indicating that the incorporation of corn stalk fibers prevented the materials from peeling off in large flakes. The micrograph in Figure 7b proved the formation of some wear debris, particles, microcracks and shallow grooves, and meanwhile, some local detached regions also presented on the worn surface, which could account for the high wear rate of specimen FC-2. The surface of specimen FC-8 was covered with fine wear debris, parallel shallow grooves and bare fibers (Figure 7e). This observation could be explained by the fact that the resin adhesiveness to corn stalk fibers decreased with the increasing fiber content, which depressed the fiber-matrix interface bonding strength, and some fibers easily fell off from the matrix under the sheer force and normal pressure. And the broken fibers and hard particles were embedded in the matrix, then scratched and damaged the surface during wear process, leading to the increase of the wear rate [45]. 

Moreover, as evident from Figure 7c,d, the specimens FC-4 and FC-6 exhibited relatively smooth worn surfaces compared with other composites. Small spalling pits, some fine wear debris and few shallow grooves as well as some apparent voids and secondary plateaus existed on the worn surface of specimen FC-4 (Figure 7c). In general, the formation of voids facilitated the absorption of braking noise to some extent, and meanwhile, some debris and particles were found in the voids, which could contribute to the reduction of the surface damage of specimen FC-4. As shown in Figure 7d, no obvious separation was found at the interface between the resin matrix and fillers, and abundant secondary plateaus presented on the surface of specimen FC-6, which were responsible for its higher wear resistance. During the wear process, the formation and development of secondary contact plateaus were attributed to the compression of wear debris at normal pressure, shear force and friction heat [46]. And the generation of secondary plateaus could induce the formation of friction film on the composite surface, which was correlated with the stable friction coefficient and small wear rate [47,48]. 

### 3.6. Analysis of Worn Surface Roughness 

The surface roughness of friction composites is significantly related to both friction behavior and wear resistance in a certain manner. An exact analysis of worn surface roughness of the friction composites was carried out by using CLSM, which enabled the three-dimensional reconstruction of surface geometry.

**Figure 8:**
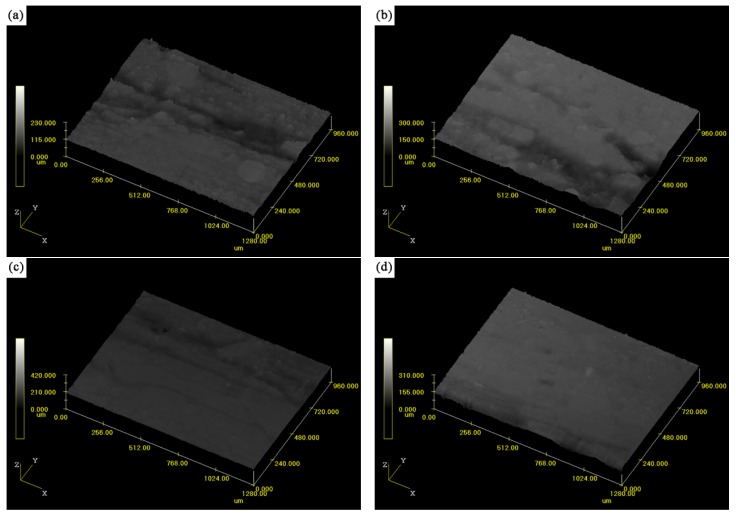

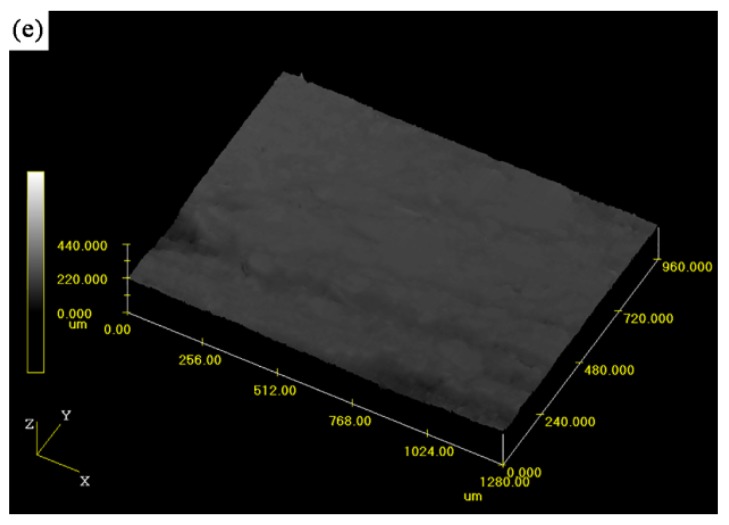
Three-dimensional reconstructions of surface geometry of (**a**) FC-0; (**b**) FC-2; (**c**) FC-4; (**d**) FC-6; (**e**) FC-8.

The main surface roughness parameters of the five friction composites, including average roughness (Ra), root-mean-square roughness (Rq), maximum valley depth (Rv) and maximum peak height (Rp), are summarized in Table 3. It can be seen that the values of Ra and Rq were in the order FC-0 > FC-2 > FC-8 > FC-4 > FC-6, which was consistent with the results of the tribological behaviors. As for the Rv and Rp values, no clear trends were observed. The Rv of the specimen FC-4 was the highest, which was attributed to the pull-out of the fibers and the formation of the cavities. The value of Rp was maximized in specimen FC-8. This may be because some wear debris piled up around the fiber ends and were compressed under the normal pressure, then the contact plateaus formed on the worn surface, which resulted in the increase of Rp. This indicated that the specimen FC-6 exhibited the lowest roughness (Ra = 1.746 μm), whereas the specimen FC-0 showed the highest roughness (Ra = 2.786 μm) among all the specimens. These results were consistent with the aforementioned tribological behavior and surface morphology analysis, as well as the reconstructed surface geometry in Figure 8. Under the condition of dry sliding, the larger roughness was mainly ascribed to the serious damage of worn surface which could cause an increase in average roughness. It suggested that the worn surface of specimen FC-6 was much smoother than other friction composites, and the specimen FC-6 possessed higher wear resistance.

## 4. Conclusions

The physio-mechanical and tribological behaviors of friction composites with different relative contents of corn stalk fibers were systematically investigated in the present study. Based on the results, the main conclusions can be summarized as follows:

1. The density and hardness of friction composites decreased with the increasing content of corn stalk fibers. At the same time, the impact strength of specimen FC-4 was the highest in comparison with that of other composites.
2. The friction coefficients of the composites generally increased first and then decreased with the temperature increase. Compared with specimen FC-0, the corn stalk fiber-reinforced friction composites showed higher friction coefficients except for the case at 100 °C.
3. The specimen FC-6 showed a fade ratio of 7.8% and recovery ratio of 106.5%, suggesting superior fade resistance and recovery performances.
4. The wear rates of all composites were significantly influenced by the test temperature and increased with temperature rise. The specimen FC-6 exhibited the lowest wear rate, except for that when the temperatures were about 100–150 °C.
5. The micrographs of worn surface morphology showed that the tribological performances of friction composites were closely associated with the formation of secondary contact plateaus on the surfaces. The specimen FC-6 had a smoother worn surface (Ra = 1.746 μm) than other friction composites and was covered with a great number of secondary plateaus and few shallow grooves, which explained the higher wear resistance.

The physio-mechanical and tribological tests confirmed that corn stalk fibers could be used as reinforcement for friction composites, which is an environmentally-friendly form of utilization of waste corn stalks.

## Figures and Tables

**Table 1:** Ingredient ratios of friction composites.

Raw Materials (by wt %)	Specimens				
	FC-0	FC-2	FC-4	FC-6	FC-8
Corn stalk fibers	0	2	4	6	8
Compound mineral fibers	25	24.42	23.84	23.26	22.68
Vermiculite powder	5	4.88	4.76	4.64	4.52
Calcium carbonate	10	9.77	9.54	9.31	9.08
Coke	5	4.88	4.76	4.64	4.52
Graphite	8	7.81	7.62	7.43	7.24
Friction powder	1	0.98	0.96	0.94	0.92
Zirconium silicate	4	3.91	3.82	3.73	3.64
Alumina	6	5.86	5.72	5.58	5.44
Barium sulfate	20	19.54	19.08	18.62	18.16
Zinc stearate	2	1.95	1.90	1.85	1.80
Phenolic resin	14	14	14	14	14

**Table 2:** Physio-mechanical properties of the friction specimens.

Specimens	Density (g·cm^−3^)	Hardness (HRR)	Impact Strength (MPa)
FC-0	2.33	103.6	0.461 ± 0.009
FC-2	2.23	101.4	0.424 ± 0.012
FC-4	2.20	98.9	0.486 ± 0.007
FC-6	2.18	97.2	0.473 ± 0.013
FC-8	2.11	95.8	0.422 ± 0.015

**Table 3:** Surface roughness parameters of the friction composites.

Specimens	Average Roughness	Root-Mean-Square Roughness	Maximum Valley Depth	Maximum Peak Height
	Ra (μm)	Rq (μm)	Rv (μm)	Rp (μm)
FC-0	2.786	3.883	71.305	34.012
FC-2	2.506	3.429	38.974	26.658
FC-4	1.838	2.661	75.024	30.059
FC-6	1.746	2.574	42.031	25.824
FC-8	2.407	3.401	48.576	35.839
